# Testing Risk-Taking Behavior in Chinese Undergraduate Students

**DOI:** 10.1371/journal.pone.0097989

**Published:** 2014-05-16

**Authors:** Xiufang Du, Jia Li, Xiulian Du

**Affiliations:** 1 School of Psychology, Shandong Normal University, Jinan, China; 2 Department of Foreign Language, Shandong University of Political Science and Law, Jinan, China; University of Granada, Spain

## Abstract

The DOSPERT, developed by Weber, Blais and Betz, can be used to measure risk behaviors in a variety of domains. We investigated the use of this scale in China. The participants were 1144 undergraduate students. After we removed some items that were not homogeneous, a principal component analysis extracted six components that accounted for 44.48% of the variance, a value similar to that obtained in the analysis conducted by Weber et al. Chinese undergraduates scored higher on the investment subscale compared with the results of Weber’s study. The analysis of individual differences indicated that there was a significant gender difference in the ethical, investment and health/safety subscales, where males scored significantly higher than females. The type of home location was also significant on the ethical and health/safety subscales, where undergraduates from the countryside scored lower than undergraduates from cities and towns on the ethical subscale, and undergraduates from towns scored higher than those from other two areas on the health/safety subscale. Male undergraduates from towns scored higher than male undergraduates from other areas on the gambling subscale.

## Introduction

Risk attitude refers to a generic orientation toward taking or avoiding a risk when deciding how to proceed in a situation with an uncertain outcome [1]. Many studies have been conducted on people’s risk-taking behaviors in different situations. These studies are mainly in two domains: decision-making and social and personality psychology. In the decision-making domain, risk attitudes are often investigated within the framework of the expected utility or its variant, prospect theory [2]. Risk attitudes are derived from a series of risky choices, such as gambling decisions. People’s risk attitudes are often described as “a descriptive label for the shape of the utility function presumed to underlie a person’s choices” (p. 264) [3]. Accordingly, risk attitudes are classified into three types: risk-seeking, risk-neutral and risk-averse. However, different results were reported with different methods in this domain, and even when the same method was used, an individual’s risk behaviors were not consistent across different situations [3]. In social and personality psychology, researchers view risk attitudes as a personality trait. Investigators use questionnaires that include items about risky behaviors to measure attitudes toward risk [1], [3], [4], [5]. These scales are designed to allow for the direct measurement of these attitudes across different domains and situations and could always obtain consistent results.

The Domain-Specific Risk-Taking (DOSPERT) scale developed by Weber et al. can be used to adequately measure risk attitudes in a variety of domains and it is based on the notion that risk-taking is content-specific. This instrument assesses risk-taking in five content domains: financial decisions (separately for investing and gambling), health/safety decisions, recreational decisions, ethical decisions and social decisions. Studies showed that the DOSPERT is valid in many other cultures such as the German, Dutch and Spanish cultures [6]. These studies all focus on the western cultural background. We know there is a great difference between eastern culture and western culture.

Risk attitudes may be influenced by culture because of its relationship to factors such as social value, social history and social ideology, which are likely to influence how people respond to risk. Indeed, cross-cultural differences have been found with regard to risky decision-making. Hsee and Weber compared the risk preferences between American and Chinese participants in decision situations that included a sure payoff option and a probabilistic payoff option [7]. The authors found that Chinese people were more risk-seeking than Americans only in the investment domain. However, there have been few cross-cultural studies of risk preference. Weber and Hsee noted that, “the past and the current levels of attention given to cultural determinants to decision-making were not just low, but inadequate” (p.34) [8].

Hu and Xie examined whether the DOSPERT scale could be used in Chinese university students [9]. They detected five main factors. Recreation, ethical, gambling and health/safety domains corresponded to Weber’s study, and social and investment items were combined. The results did not replicate the findings reported by Weber et al. [3]. It is possible however that the design of this study may have influenced the results. For example, the sample size was small and the participants all come from the same university. This study also did not report the total variance explained by each factor, nor did they collect data to assess test-retest reliability. Therefore, the validity of this result is questionable. The validity and reliability of the DOSPERT scale in a Chinese cultural context is in need of further research.

Many researchers suggest that gender is an important factor that influences risk attitudes: women are less willing to take risks than men [3], [5], [10]. Weber et al. found gender differences in all areas except the social domain [3]. Harris, Jenkins and Glaser assessed undergraduates’ likelihood of engaging in various risky activities [11]. They also found that in the health, recreational and gambling domains, women reported a lower likelihood of engaging in risky behaviors. A meta-analysis by Byrnes, Miller and Schafer reviewed over 150 papers on gender differences in risk perception [12]. They concluded that the literature indicated that men take more risks than women overall. Age is also a stable factor that influences the attitude toward risk: Increasing age is associated with a decreased risk-taking [10], [13]. As for the family background, Dohmen et al. found that individuals with highly educated parents were more willing to take risks [10]. However, others have reported that the influence of this factor is not stable [14], [15]. In Chinese society, there is a large difference between urban and rural areas with regard to economics, education level, parental rearing styles and other factors. The second purpose of our study is to explore how individual differences (such as gender, and home location) are related to the risk attitudes of Chinese undergraduate students.

## Method

### Participants

A total of 1,227 undergraduates volunteered to take part in this study. The participants were recruited at separate times. The first sampling is designated as sample one, and the second sampling is designated as sample two. The participants in sample one were from three universities: Shandong Normal University, Shandong Jianzhu University and University of Jinan. After getting in touch with the university student affairs offices, 604 students agreed to participate in our survey. Of all of the responses, 550 were valid, and the rest of them were incomplete (more than 30% items unanswered). Sample one was used for item analysis and principal components analysis. This sample comprised 244 males and 216 females, 218 majors in humanities and arts and 332 majors in science, 121 people from cities, 138 from towns and 291 from the countryside. Their average age was 19.35, ranging from 17 to 25. The participants in sample two were from Shandong Normal University, Shandong University of Science and Technology and Shandong Institute of Education. Altogether, 623 students participated in this survey. After excluding the incomplete questionnaires, 594 responses were valid. Sample two was used for confirmatory factor analysis and individual difference analysis. This sample comprised 196 males and 398 females, 358 majors in humanities and arts and 236 majors in science, 159 people from cities, 196 from towns and 239 from the countryside. Their average age was 19.26, ranging from 17 to 26.

Ethical approval for this study was obtained from the Academic Board of Shandong Normal University. This institutional academic committee is responsible for ethical review. Before we began this investigation, we informed the students that the survey was completely voluntary and that the data would be analyzed anonymously. The students were informed that they could refuse participation. Completing the questionnaire was considered as “implied consent.”

### Materials

#### The domain specific risk-taking scale

The DOSPERT scale was designed by Weber et al. [3]. It consists of 40 items in the financial, health/safety, recreational, ethical and social domains. Because the financial domain comprises two dimensions (investing and gambling), the scale is actually best regarded as comprising six dimensions. Cronbach’s alpha typically ranges between 0.71 and 0.86. The scale can be used to measure risk behavior, risk perception and perceived benefits. For risk behavior, the respondents evaluate their likelihood of engaging in specific risky behaviors on a five-point rating scale ranging from 1 (“Extremely unlikely”) to 5 (“Extremely likely”). For risk perception, the participants rate their perception of the risk entailed by each risky behavior on a five-point rating scale ranging from 1 (“Not at all risky”) to 5 (“Extremely risky”). For perceived benefits, the participants indicate the benefits they would obtain from each situation on a five-point rating scale ranging from 1 (“No benefits at all”) to 5 (“Great benefits”). In this study, we focused on risky behavior.

#### Sensation-seeking scale

Zuckerman’s Sensation-Seeking Scale (SSS) form V has been translated by Wang [16], [17]. It contains 40 forced-choice items, such as: “I sometimes like to do things that are a little frightening.” The form contains four subscales: thrill- and adventure-seeking (TAS), experience- seeking (ES), disinhibition (DIS) and boredom susceptibility (BS). Each subscale is represented by 10 items. The three-week test-retest reliabilities of these four subscales have been reported within the range 0.70–0.94, and the internal consistency reliability is within the range 0.58–0.82 [15].

### Procedure

The English version of the DOSPERT scale was independently translated by four psychology graduates. They then cross-checked their translations with each other, and they made corrections together. This ensured that the original meaning would be maintained and that every item would be represented appropriately in the Chinese language. After that, a graduate student with a major in English and a graduate student studying in America translated the Chinese version back into English. We found minimal differences between the resulting English version and the original version. Thus, the Chinese version corresponded semantically to the English version and was suitable for our purposes. One item, “Betting a day’s income at the horse races,” was changed to “Betting a day’s income in the lottery” because betting on horse races is not legal in mainland China.

A total of 1,144 participants composing two samples took part in the survey. In addition, 155 participants from sample two were measured again four weeks later. They were all selected from the School of Psychology of Shandong Normal University by cluster sampling. SPSS13.0 and AMOS7.0 for Windows were used to analyze the data.

## Results

### Psychometric Analysis of the Chinese Version of the DOSPERT Scale

#### Preliminary item analysis

We first conducted item-total correlations for discriminating the items in Sample One. The corrected item-total correlations for item 1 (“Admitting that your tastes are different from those of your friends”), item 23 (“Telling a friend if his or her significant other has made a pass at you”) and item 34 (“Taking a job that you enjoy over one that is prestigious but less enjoyable”) were 0.084, 0.038 and 0.148 respectively. A correlation value less than 0.2 indicates that the corresponding item does not correlate very well with the scale overall, and thus, items 1, 23 and 34 should be dropped [18].

Items were examined again in terms of internal consistency using the Cronbach’s alpha approach. The same results were obtained. The Cronbach’s alpha of 40 item scale was 0.862. When item 1 was deleted, the Cronbach’s alpha increased to 0.864. When item 23 was deleted, the Cronbach’s alpha increased to 0.865. When item 34 was deleted, the Cronbach’s alpha increased to 0.863. However, if we deleted other items, the Cronbach’s alpha did not increase. Therefore, items 1, 23 and 34 were excluded from the factor analysis.

#### Principal components analysis

The Kaiser-Meyer-Olkin (KMO) value measure of sampling adequacy was 0.86, and Bartlett’s test of sphericity was χ^2^ = 4864.57, *p*<0.001. These values indicate that the data were suitable for principal component analysis (PCA).

Principal component analysis (PCA) and varimax rotation were used to investigate the factor structure of the Chinese version of the DOSPERT. We extracted six components. The results showed that most of the items loaded on dimensions similar to those in Weber’s model (2002). However, three items were associated with dimensions different from those that were associated with in the original model. Item 4 (“Buying an illegal drug for your own use”) loaded on the gambling dimension, but it had originally been loaded on the health/safety dimension. Item 5 (“Cheating on an exam”) loaded on the health/safety dimension, but it had originally loaded on the ethical dimension. Item 27 (“Engaging in unprotected sex”) loaded on the ethical dimension, but it had originally loaded on the health/safety dimension. To maintain consistency with the original scale, items 4, 5 and 27 were therefore excluded. The 34 items that were retained accounted for 44.48% of the variance.

The model’s factor structure is shown in Table 1. Items 2, 6, 15, 17, 21, 31, 37 and 38 related to recreational risk loaded highly on component 1 (“recreational risk,” which accounted for 17.80% of the variance). Items 9, 12, 13, 14, 20, 25 and 28 loaded on component 2 (“ethics,” which accounted for 8.77% of the variance). Component 3 consisted of items 7, 18, 24 and 30 (“investment behaviors,” which accounted for 5.34% of the variance). Items 3, 11, 22 and 33 loaded on component 4 (“gambling behavior,” which accounted for 4.57% of the variance). Component 5 (items 10, 16, 19, 26 and 35) and component 6 (items 8, 29, 32, 36, 39 and 40) explained 4.26% and 3.73% of the variance as social risk and health/safety risk, respectively.

**Table 1:** The DOSPERT Items and Principal-Component Loadings.

Items	Loadings						
	1	2	3	4	5	6	CFA
21 Going whitewater rafting during rapid water flows in the spring	**0.70**	0.12	0.11	0.00	−0.05	0.08	0.50
17 Going down a ski run that is beyond your ability or closed	**0.65**	0.09	0.00	0.15	0.14	0.06	0.53
31 Periodically engaging in a dangerous sport (e.g., mountain climbing or sky diving)	**0.64**	0.06	−0.01	0.14	0.12	0.06	0.60
37 Trying out bungee jumping at least once	**0.55**	−0.09	0.28	−0.07	0.16	0.08	0.45
15 Going on a vacation in a third-world country without prearranged travel and hotel accommodations	**0.54**	0.18	0.14	0.06	0.22	−0.05	0.44
6 Chasing a tornado or hurricane by car to take dramatic photos	**0.54**	0.24	0.05	0.19	−0.07	0.14	0.52
2 Going camping in the wilderness, beyond the civilization of a campground	**0.49**	0.15	−0.16	0.11	0.23	0.11	0.52
38 Piloting your own small plane, if you could	**0.46**	0.03	0.24	0.02	0.26	0.13	0.48
14 Passing off somebody else’s work as your own	0.09	**0.73**	0.10	0.01	−0.05	0.22	0.53
28 Stealing an additional TV cable connection off the one you pay for	0.05	**0.68**	−0.11	0.21	0.11	−0.09	0.53
13 Forging somebody’s signature	0.14	**0.65**	0.11	−0.03	0.02	0.21	0.59
12 Having an affair with a married man or woman	0.21	**0.63**	0.11	0.07	0.01	0.09	0.57
25 Shoplifting a small item (e.g., a lipstick or a pen)	−0.04	**0.62**	−0.17	0.29	0.13	−0.19	0.52
20 Illegally copying a piece of software	0.27	**0.42**	0.07	0.17	0.02	0.33	0.53
9 Cheating by a significant amount on your income tax return	0.25	**0.39**	0.05	0.31	0.17	−0.08	0.54
24 Investing 5% of your annual income in a conservative stock	0.02	−0.08	**0.81**	0.00	0.02	0.05	0.72
30 Investing 10% of your annual income in government bonds (treasury bills)	0.11	0.00	**0.71**	0.03	0.10	0.08	0.58
7 Investing 10% of your annual income in a moderate growth mutual fund	−0.02	0.09	**0.67**	0.02	0.30	−0.04	0.54
18 Investing 5% of your annual income in a very speculative stock	0.25	0.15	**0.56**	0.15	0.09	0.00	0.61
3 Betting a day’s income at the lottery	0.06	−0.11	0.10	**0.70**	0.05	0.02	0.51
11 Betting a day’s income at a high-stakes poker game	0.11	0.21	0.07	**0.70**	−0.07	0.08	0.71
22 Betting a day’s income on the outcome of a sporting event (e.g., baseball, soccer or football)	0.13	0.21	0.05	**0.60**	0.03	0.13	0.67
33 Gambling a week’s income at a casino	0.01	0.39	−0.16	**0.60**	−0.02	−0.06	0.61
35 Defending an unpopular issue that you believe in at a social occasion	0.20	0.08	−0.02	−0.07	**0.65**	−0.05	0.37
16 Arguing with a friend about an issue on which he or she has a very different opinion	0.05	−0.04	0.12	−0.11	**0.60**	0.09	0.51
10 Disagreeing with your father on a major issue	0.06	0.05	0.07	0.04	**0.50**	0.09	0.37
19 Approaching your boss to ask for a raise	0.06	−0.02	0.27	0.05	**0.44**	0.07	0.45
26 Wearing provocative or unconventional clothes on occasion	0.24	0.06	0.10	0.08	**0.41**	0.12	0.46
29 Not wearing a seatbelt when riding as a passenger in the front seat	−0.05	0.08	0.09	0.07	0.21	**0.66**	0.41
32 Not wearing a helmet when riding a motorcycle	0.21	0.10	0.05	0.17	−0.01	**0.58**	0.58
40 Regularly eating high cholesterol foods	0.05	0.12	−0.02	−0.03	0.06	**0.58**	0.42
36 Exposing yourself to the sun without using sunscreen	0.12	−0.10	0.00	−0.06	0.38	**0.45**	0.31
39 Walking home alone at night in a somewhat unsafe area of town	0.37	0.11	−0.09	0.09	0.30	**0.39**	0.47
8 Consuming five or more servings of alcohol in a single evening	0.23	0.06	0.14	0.35	−0.07	**0.36**	0.47
Account(%)	17.80	8.77	5.34	4.57	4.26	3.73	
Accumulated contribution rate(%)	17.80	26.58	31.92	36.49	40.75	44.48	

#### Confirmatory factor analysis

To verify the current exploratory 34-item model,a confirmatory factor analysis was conducted using AMOS7.0 based on the data from sample two. Results of the CFA showed that the data fitted the six-factor model well (χ2/df = 1.18, root mean square error approximation (RMSEA) = 0.02, comparative fit index (CFI) = 0.97, Tucker-Lewis index (TLI) = 0.98, adjusted goodness-of-fit index (AGFI) = 0.96). The standard regression weights of the items on the risk-taking scale ranged from 0.31 to 0.72.

#### Reliability

A total of 155 participants were administered the DOSPERT scale on two occasions separated by a four-week interval. The test-retest reliability of the total risk attitude scale and subscale was assessed using intra-class correlation. These analyses yielded coefficients of 0.49–0.81 for the subscales and 0.77 for the total scale (see Table 2). For the gambling subscale, the test-retest reliability was relatively low (0.49).

**Table 2:** Reliability of the Risk-Taking Scale.

	Recreational	Ethical	Investment	Gambling	Social	Health/safety	Total Scale
Homogeneity reliability(n = 594)	0.73	0.76	0.70	0.72	0.51	0.59	0.86
Test-retest reliability(n = 155)	0. 81	0.60	0.72	0.49	0.70	0.70	0.76

An additional statistic, homogeneity reliability, was extracted from the data from sample two, and it yielded alpha coefficients of 0.51–0.76 for the subscales and 0.86 for the total scale (see Table 2). For the social subscale, the homogeneity reliability was relatively low (0.51).

#### Criterion-related validity

Sensation-seeking, as defined by Zuckerman [19], is “…the need for varied, novel and complex sensations and experiences and the willingness to take physical and social risks for the sake of such experiences” [20]. This parameter is a good predictor of risk behavior [21], [22]. Using the sensation-seeking scale as a validity criterion, we conducted a correlation analysis with the risk-taking scale and the sensation-seeking scale (Table 3). As predicted, sensation-seeking (and its various subscales) correlated significantly with the risk-taking subscales in all six dimensions.

**Table 3:** Pearson Correlations between Risk-Taking Subscales and Sensation-Seeking Subscales.

	DOSPERT	Recreation	Ethical	Investment	Gambling	Social	Health/safety
TAS	0.42**	0.59**	0.09*	0.10*	0.20**	0.22**	0.26**
ES	0.34**	0.43**	0.11*	0.10*	0.16**	0.28**	0.17**
DIS	0.40**	0.34**	0.32**	0.18**	0.20**	0.22**	0.26**
BS	0.32**	0.34**	0.24**	0.10*	0.13**	0.15**	0.20**
SS	0.51**	0.61**	0.24**	0.16**	0.24**	0.30**	0.31**
Recreational	0.80**	1.00					
Ethical	0.69**	0.37**	1.00				
Investment	0.56**	0.33**	0.41**	1.00			
Gambling	0.54**	0.36**	0.22**	0.21**	1.00		
Social	0.57**	0.35**	0.25**	0.20**	0.28**	1.00	
Health/safety	0.69**	0.45**	0.35**	0.25**	0.22**	0.34**	1.00

Note 1: *p<0.05,**p<0.01,***p<0.001;

Note 2: TAS: thrill- and adventure-seeking, ES: experience-seeking, DIS: disinhibition, BS: boredom susceptibility.

### Individual Difference Analysis

Table 4 shows the subscale means and standard deviations for different genders and different home locations. We performed a 2 (gender: male vs. female) × 3 (location: city, town vs. countryside) multivariate analysis of variance to test the differences of gender and location on risk-taking. The results showed that the main effects of gender in the ethical, investment and health/safety subscales were significant (*F*(1,588) = 13.10, *p*<0.001; *F*(1,588) = 7.22, *p*<0.01; *F*(1,588) = 24.97, *p*<0.001), where males scored significantly higher than females. The main effects of location on the ethical and health/safety categories were significant (*F*(2,588) = 7.19, *p*<0.001; *F*(2,588) = 6.49, *p*<0.01). Pairwise comparisons with home location showed that undergraduates from the countryside scored significantly lower than undergraduates from cities and towns on the ethical subscale (*ps*<0.01) and that undergraduates from towns scored significantly higher than those from towns and countryside on the health and safety subscale (*ps*<0.05). We also found a significant interaction between gender and location in the gambling subscale, *F*(2,588) = 3.54, *p*<0.05. Analyses of the simple main effects revealed that the difference of home location was significant only for males, *F*(2,589) = 4.42, *p*<0.05. Pairwise comparisons with home location showed that male undergraduates from towns scored higher than male undergraduates from cities and countryside on the gambling subscale (*p*s<0.05); no other significant differences were found.

**Table 4:** Means and Standard Deviations for the Different Samples in Risk-Taking.

		Recreational		Ethical		Investment		Gambling		Social		Health/Safety	
		M	SD	M	SD	M	SD	M	SD	M	SD	M	SD
Male	City	2.90	0.68	2.01	0.58	3.72	0.97	1.47	0.60	3.59	0.36	3.03	0.62
	Town	3.03	0.58	2.10	0.69	3.95	0.64	1.86	0.83	3.81	0.52	3.56	0.62
	Countryside	2.94	0.65	1.76	0.70	3.73	0.81	1.45	0.52	3.64	0.55	3.27	0.49
Female	City	2.82	0.73	1.78	0.66	3.55	0.74	1.55	0.67	3.65	0.54	2.82	0.69
	Town	2.90	0.66	1.78	0.55	3.56	0.69	1.48	0.64	3.73	0.48	2.92	0.61
	Countryside	2.75	0.68	1.63	0.55	3.60	0.64	1.53	0.60	3.68	0.55	2.95	0.66

## Discussion

The results presented here demonstrate the validity of the DOSPERT in a Chinese population. The factor structure identified by Weber et al. in a sample of American college students was replicated. Our principal component analysis also detected six components that corresponded well to Weber’s study: recreation, ethical, investment, gambling, social and health/safety domains. This was not consistent with Hu and Xie’s study [9], which detected five main factors. Recreation, ethical, gambling and health/safety domains corresponded to Weber’s study, but social and investment items were combined. Our six-factor model accounted for 44.48% of the variance, which is a bit lower than the value obtained in the analysis conducted by Weber et al. (see study 3) [3].

However, in this study, six items were deleted. Items 1, 23 and 34 were deleted because of their poor ability to discriminate. Items 4, 5 and 27 were deleted because the dimensions with which they were associated were not those that were theoretically expected. These discrepancies might reflect different cultural values. For example, consider item 27 (“Engaging in unprotected sex”). In Chinese culture, people are conservative in the way that they regard sexual behavior; they view sex as an ethical issue rather than as a matter of health or safety. For item 4 (“Buying an illegal drug for your own use”), the ban on illegal drugs is strictly enforced by the Chinese government. Very few Chinese university students can come into contact with drugs. For item 5 (“Cheating on an exam”), cheating on an exam used to be very common in Chinese university campuses in their early years. Currently, however, most universities have taken cheating seriously. Once a student is found cheating on exams, he or she will be severely punished. Chinese university students may not consider cheating on an exam as an ethical question. We also do not believe that cheating on an exam is a health/safety question, although it loaded on the health/safety dimension. Except for the six items above, all 34 items loaded on the target factors were identical to those in the study by Weber et al. [3].

In this study, the homogeneity reliability of the total scale is 0.86, similar to Weber’s 0.88, and 0.51–0.76 for the subscales. The social subscale was the least reliable (0.51). As to the test-retest reliability, the gambling subscale was the least reliable (0.49). The test-retest reliability of the subscale was 0.49–0.81 for the subscales, respectively, and 0.77 for the total scale (see table 2). Criterion related validity analysis resulted that all subscales showed moderate positive correlations with subscales of the sensations-seeking scale. CFA showed that the indexes used to determine the goodness of fit are satisfactory. CFA showed that all of the item loadings were above 0.31. All of these loadings indicated that the Chinese scale is valid and reliable. The correlations among the factors ranged from 0.20 (between social and investment factors) to 0.45 (between health/safety and recreation factors), with an average correlation among factors of 0.30. Given that all of the DOSPERT factors were significantly correlated, it is more sensible to use the common methods of oblique rotation in PCA. In the CFA, however, we have confirmed the factor structure, so we believe that the use of a varimax rotation did not influence the results.

On the investment subscale (it consists of the same items as Weber’s), unlike the results obtained by Weber et al. for a sample of American college students (males = 2.75,females = 2.38) [3], Chinese undergraduate students scored higher (males = 3.81,females = 3.57). The differences are significant (t_male_ = 10.34, *p*<0.001; t_female_ = 18.52, p<0.001). On other subscales, however, the average scores are similar to those in Weber’s study. We can argue that with respect to investment, people in China are more risk-seeking. This can be seen in Hsee and Weber’s study [7]. In that experiment, the participants were asked to choose between a sure payoff option (savings) and a probabilistic payoff option (stock). The results showed that Chinese participants were more risk-seeking than Americans in the investment domain. They explained the result in terms of a “cushion hypothesis.” According to this view, members of socially collectivist cultures, such as the Chinese culture, can afford to take greater financial risks because they have larger social networks than people from individualist cultures. The social network serves as a cushion that can protect them against catastrophic outcomes [7].

The well-known gender difference in risk behavior also holds true within the Chinese culture. Our parallel results previously reported significant male-female differences in risk-taking attitudes, with female respondents being less likely to engage in risky behaviors. Hu and Xie also found male respondents to the DOSPERT were more risk-taking than female participants in all domains except the social domain [9].

In our results, significant gender differences were observed in the ethical, investment and health/safety subscales. Males were more risk-seeking in these domains than females. Men also scored higher than women on the recreational subscale, but the difference was not significant. In contrast, women scored higher than men in the social domain, although, again, the difference was not significant. These findings broadly replicate the results obtained by Weber et al. [3], who found gender differences in all areas except the social domain. Similar gender differences have been found in these same domains in a large German sample [6]. In the German sample, as in our Chinese sample, gender differences in social risk-taking were not found. Additionally, in the four different domains (gambling, health, recreational and social), Harris, Jenkins and Glaser did not find gender differences in undergraduates’ likelihood of engaging in socially risky activities [11].

Why do gender differences exist in risky behaviors across many domains? Two explanations are possible. One is the Darwinian analysis of parental investment suggested by Buss [11]. For physiological reasons, the minimum investment required to produce offspring is much greater for females than for males. There is much greater variability in male reproductive success than in female reproductive success. Therefore, males may be willing to take greater risks for a chance to increase their attractiveness to mates. Li and Zhang’s experiment supported this hypothesis [23]. The authors explored the influence of mating and reward cues on the risk behaviors of males and females using an implicit priming method. Their results showed that men were more likely to take risks after being exposed to attractive opposite-sex pictures than to reward pictures, whereas women did not show this difference. These results support the notion that men’s risk-taking is driven by mating motivation. The second explanation is also a biological one. Although some studies reported mixed or null effect [24], on the whole, many studies suggested a positive relationship between an individual’s risk behaviors and testosterone levels in his or her body [25], [26] [27]. As a result, men are more likely to take risks than women, especially in the ethical, gambling and health/safety domains.

In addition to these two accounts, we believe that gender differences are also environmental. Child-rearing patterns, socialization and sex role expectations can partly explain these gender differences. In many societies, men are encouraged to take more risks than women in certain areas. Parents may think that some risky activities (such as drinking alcohol) could benefit boys in the long run. Therefore, most of the time, they encourage boys to engage in certain risks, but girls are discouraged from them. As they grow, boys are expected to assume responsibility, seek adventure and exhibit bravery and independence, and girls are considered to be tender, timid and dependent, especially in male-dominated cultures.

Our results showed that the risk behaviors of students from different areas were significantly different in the ethical and health/safety domains. The undergraduates from the countryside were more risk-averse with regard to ethical matters than students from other places. Evidence from the scientific literature suggested that family influences and media exposure may affect individual risk behaviors [28], [29], [30]. This result may reflect the fact that students from rural areas have been more strongly influenced by their parents from their early childhood to obey conservative ethical rules. The undergraduates from towns were more risk-seeking with regard to health and safety matters than those from cities and countryside, and male undergraduates from towns scored higher than male undergraduates from cities and countryside on the gambling subscale. These findings indicate that in China, town is a special area where urban civilization and rural civilization are in fierce collision. Undergraduates from towns who are deeply influenced by rural civilization have strong yearning for urban civilization. On the other hand, the economic level of the town is better than the countryside, while they both have a gap compared with the city. There are certain resources and opportunities in the town. Therefore, to some extent, for people in the town (especially for male), taking more risks means more opportunities. So it is easy to explain why undergraduates from towns were more risk-seeking with regard to health/safety and gambling matters than those from cities and the countryside. We also can see that undergraduates from towns scored higher in all other dimensions than those from other areas (see Table 4), though the differences were not significant.

This study has some advantages. We have examined the factor structure of the DOSPERT in a large Chinese sample. The result showed that the Chinese version replicated many of the important findings obtained by Weber et al. [3]. The study also systematically examined individual difference factors such as gender and home location related to the risk-taking subscales in China. An explanation was also provided according to Chinese culture. Nevertheless, this study has some limitations that should be noted. First, the six factors only accounted for 44.48% of the variance. This is somewhat lower than those reported by Weber et al. [3]. Secondly, the homogeneity reliability was relatively low for the social subscale, as was the test-retest reliability for the gambling subscale, which indicated that items in these domains may need further improvement in future research. Finally, this study only examined the validity of the risk-taking scale in Chinese students, not involving the risk-perception scale.

In conclusion, this study demonstrated the reliability and validity of the DOSPERT scale in a Chinese population. Therefore, the Chinese version of the DOSPERT can be used to study individual risk behaviors in China. However, the reliability of the social subscale and the gambling subscale requires further improvement. Compared with American culture, based on the study by Weber et al., Chinese undergraduate students scored higher on the investment subscale. The analysis of the individual difference indicated a significant gender difference in the ethical, investment and health/safety scores. The difference related to home location was significant for the ethical and health/safety scores.
